# Comparative transcriptome analysis of the Pacific White Shrimp (*Litopenaeus vannamei*) muscle reveals the molecular basis of residual feed intake

**DOI:** 10.1038/s41598-017-10475-y

**Published:** 2017-09-05

**Authors:** Ping Dai, Sheng Luan, Xia Lu, Kun Luo, Jie Kong

**Affiliations:** 1 Key Laboratory for Sustainable Utilization of Marine Fisheries Resources, Ministry of Agriculture, Yellow Sea Fisheries Research Institute, Chinese Academy of Fishery Sciences, Qingdao, 266071 China; 2 Laboratory for Marine Fisheries Science and Food Production Processes, Qingdao National Laboratory for Marine Science and Technology, Qingdao, 266235 China

## Abstract

Feed efficiency is an economically important trait in genetic improvement programs of *L. vannamei*. Residual feed intake (RFI), an ideal measure of feed efficiency, is the difference between observed feed intake and expected feed requirement predicted from maintenance and production. Exploring the molecular basis of RFI is essential to facilitate the genetic breeding of feed efficiency in *L. vannamei*. However, few studies have been reported in this aspect. In this study, we sequenced muscle transcriptomes of a high﻿-efficiency group, a low﻿-efficiency group and a control group originating from two families, and compared the gene expression patterns between each extreme group and the control group. A total of 383 differentially expressed genes were identified, most of which were involved in cell proliferation, growth and signaling, glucose homeostasis, energy and nutrients metabolism. Functional enrichment analysis of these genes revealed 13 significantly enriched biological pathways, including signaling pathways such as PI3K-Akt signaling pathway, AMPK signaling pathway and mTOR signaling pathway, as well as some important pathways such as ubiquitin mediated proteolysis, cell cycle, pentose phosphate pathway and glycolysis/gluconeogenesis. These genes and pathways provide initial insight into the molecular mechanisms driving the feed efficiency in *L. vannamei*.

## Introduction

The Pacific white shrimp (*Litopenaeus vannamei*) is one of the primary aquaculture species worldwide^1^. Feed cost accounts for 50–60% of total production costs in intensive shrimp culture^2^. Thus, improving profitability of production could be achieved by reducing feed cost without sacrificing production, or increasing the efficiency of feed utilization. Feed efficiency is becoming an economically important trait in genetic improvement programs of *L. vannamei*. Regarding the measure of feed efficiency, residual feed intake (RFI) has been increasingly recognized as better than a ratio trait such as feed efficiency ratio (FER)^3, 4^. RFI is defined as the difference between actual feed intake and predicted feed intake based on the requirements for maintenance of body weight and production^5^. A low value of RFI stands for a high feed efficiency, while a high value indicates a low feed efficiency. Genetic association between energy requirement for maintenance of body weight and RFI has been reported in some species^6, 7^. Understanding the genetic basis of RFI at the molecular level is expected to shed light on genetic breeding of feed efficiency in *L. vannamei*.

Obviously, RFI is a typical quantitative trait, which is characterized by complex interactions between cellular constituents such as DNA, RNA and proteins and affected simultaneously by multiple biological processes^8^. It is estimated in cattle that basic metabolic processes including protein turnover, tissue metabolism and stress response account for at least 37% of the variation in RFI and other sources of variation such as body composition, digestion, physical activity and thermoregulation each explains 5–10%^9^. Hundreds of genes and genetic markers associated with RFI have been reported in terrestrial agricultural animals such as cattle^10–13^, pig^14, 15^ and chicken^16, 17^. The knowledge of gene functions and interactions will provide further insight into the molecular mechanisms underlying RFI phenotype. To date, few studies have analyzed RFI at the molecular level in *L. vannamei* mainly because of lacking its whole genome sequence information.

The emergence and development of high-throughput sequencing technologies have fulfilled the molecular investigation of species with complex genomes. Among these technologies, the well-developed RNA sequencing (RNA-seq) allows rapid and comprehensive understanding of transcriptome level of variations^18^, and provides valuable information of gene function, cell responses and evolution^19, 20^, which applies to non-model organisms without reference genomes in particular. Using RNA-seq, for example, significant progress has been made in uncovering the expression profiles of various marine crustacea such as *L. vannamei*, *Fenneropenaeus chinensis*, *Eriocheir sinensis* and *Macrobrachium nipponense* ^21–24^.

The goal of this study was to identify genes associated with RFI and to reveal the biological processes and mechanisms determining the RFI variation. Muscle tissue is a major component of shrimp, so it is expected to be an ideal subject for investigating the molecular basis of RFI in *L. vannamei*. In this study, we chose a family with the highest RFI and one with the lowest RFI from 33 families after a feeding test. Then we set up a high-efficiency group, a low-efficiency group and a control group, from the individuals of the two families based on their RFI values. By sequencing and comparing the transcripts of muscle samples between the two groups with extreme RFI and control group, we sought to acquire abundant information about the molecular basis of RFI variation in *L. vannamei*.

## Results

### Analysis of traits

The average values of average daily gain (ADG), daily feed intake (DFI) and FER of all test animals were 0.129 ± 0.035 g/day, 0.194 ± 0.040 g/day and 0.675 ± 0.162, respectively. The coefficients of variation for ADG, DFI and FER were 27.2%, 20.4% and 23.9%, respectively, indicative of large inter-individual variations in these traits.

The model for estimating RFI was:

$${\rm{DFI}}=0.067\times {{\rm{MW}}}^{0.454}+0.394\times {\rm{ADG}}+{\rm{e}}$$

**Figure 1:**
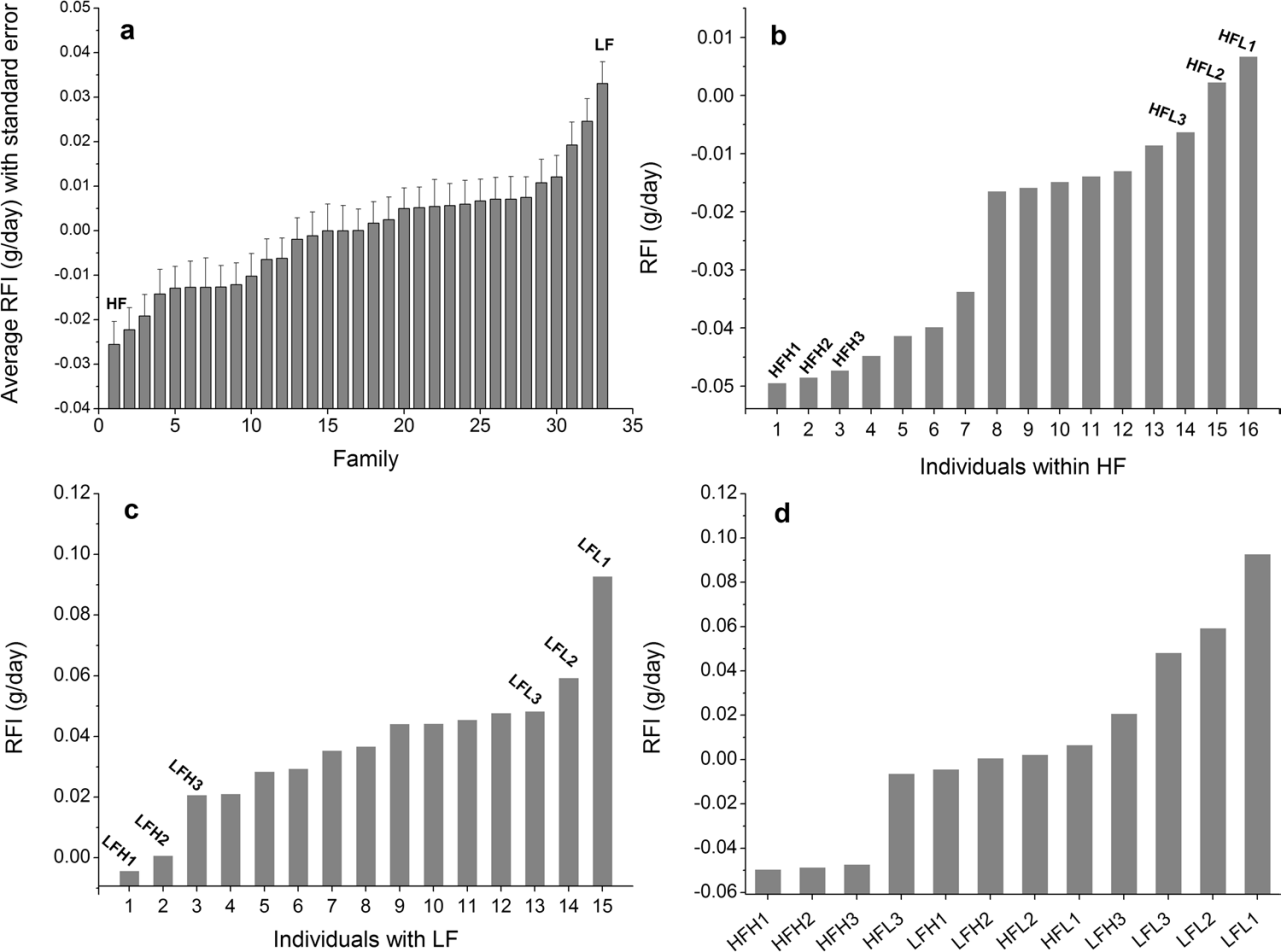
RFI distribution of test families and candidate individuals. (**a**) RFI distribution of 33 test families. (**b**) RFI distribution of individuals within the family with the highest feed efficiency (HF). The three most efficient individuals (HFH1, HFH2 and HFH3) and the three least efficient individuals (HFL1, HFL2 and HFL3) are indicated. (**c**) RFI distribution of individuals within the family with the lowest feed efficiency (LF). The three most efficient individuals (LFH1, LFH2 and LFH3) and the three least efficient individuals (LFL1, LFL2 and LFL3) are also indicated. (**d**) RFI distribution of the 12 individuals used for RNA-seq.

Estimated RFI values of test animals ranged from −0.068 to 0.092 g/day. The average RFI of the 33 families showed a range of −0.026 to 0.033 g/day (Fig. 1a). Obvious inter-individual variations in RFI were observed within the most efficient family (HF; Fig. 1b) and within the least efficient family (LF; Fig. 1c), respectively. Within each family, the three most efficient individuals (HFH1, HFH2 and HFH3 for HF, and LFH1, LFH2 and LFH3 for LF) and the three least efficient individuals (HFL1, HFL2 and HFL3 for HF, and LFL1, LFL2 and LFL3 for LF) used for RNA-seq are indicated. There were also considerable variations in RFI among the 12 individuals (Fig. 1d).

### *De novo* assembly of the transcriptome

Illumina sequencing of 12 muscle samples and one hepatopancreas sample totally generated 680,960,100 raw reads, with an average read length of 150 bp. As a benchmark for sequencing quality, values of the Q30 percentage ranged from 95.93% to 96.93%. Raw read data are archived in the NCBI Sequence Read Archive (SRA) browser (Bioproject accession number: SRR5134062 and SRR5135715). The trimmed and quality-filtered reads from all the libraries were assembled *de novo* into 72,120 unigenes, whose lengths ranged from 201 bp to 38,364 bp, with an average length of 1,484 bp and median size (N50) of 2,841 bp. Among these unigenes, 47,509 genes had a length more than 500 bp and 29,446 had a length more than 1000 bp. The total length of the unigenes was up to 107,047,713 bp.

### Functional annotation of unigenes

All the 72,120 unigenes were used as queries for BLAST searches against the NCBI non-redundant (Nr) protein database, the Swiss-Prot protein database, the Gene Ontology (GO) database, and the Kyoto Encyclopedia of Genes and Genomes (KEGG) database, respectively, with E-values ≤ 1E-5 for gene identity. As summarized in Table 1, the highest percentage of genes (32.95%) were annotated in the Nr database, followed by 27.57% matched to the Swiss-Prot database and by 25.64% matched to the GO database. The lowest percentage of genes (10.73%) were annotated in KEGG database, and specifically, 7,742 unigenes were assigned to 271 different pathways (Supplementary Table S1). Only 7,014 unigenes (9.72%) were annotated in all of the four databases.

**Table 1:** Summary statistics of annotations for the unigenes assembled *de novo*.

Category	Number of unigenes	Annotation ratio (%)
Annotated in Nr	23,761	32.95
Annotated in Swiss-Prot	19,886	27.57
Annotated in GO	18,490	25.64
Annotated in KEGG	7,742	10.73
Annotated in all above databases	7,014	9.72
Total Unigenes	72,120	100

### Cluster analysis and individual grouping

**Figure 2:**
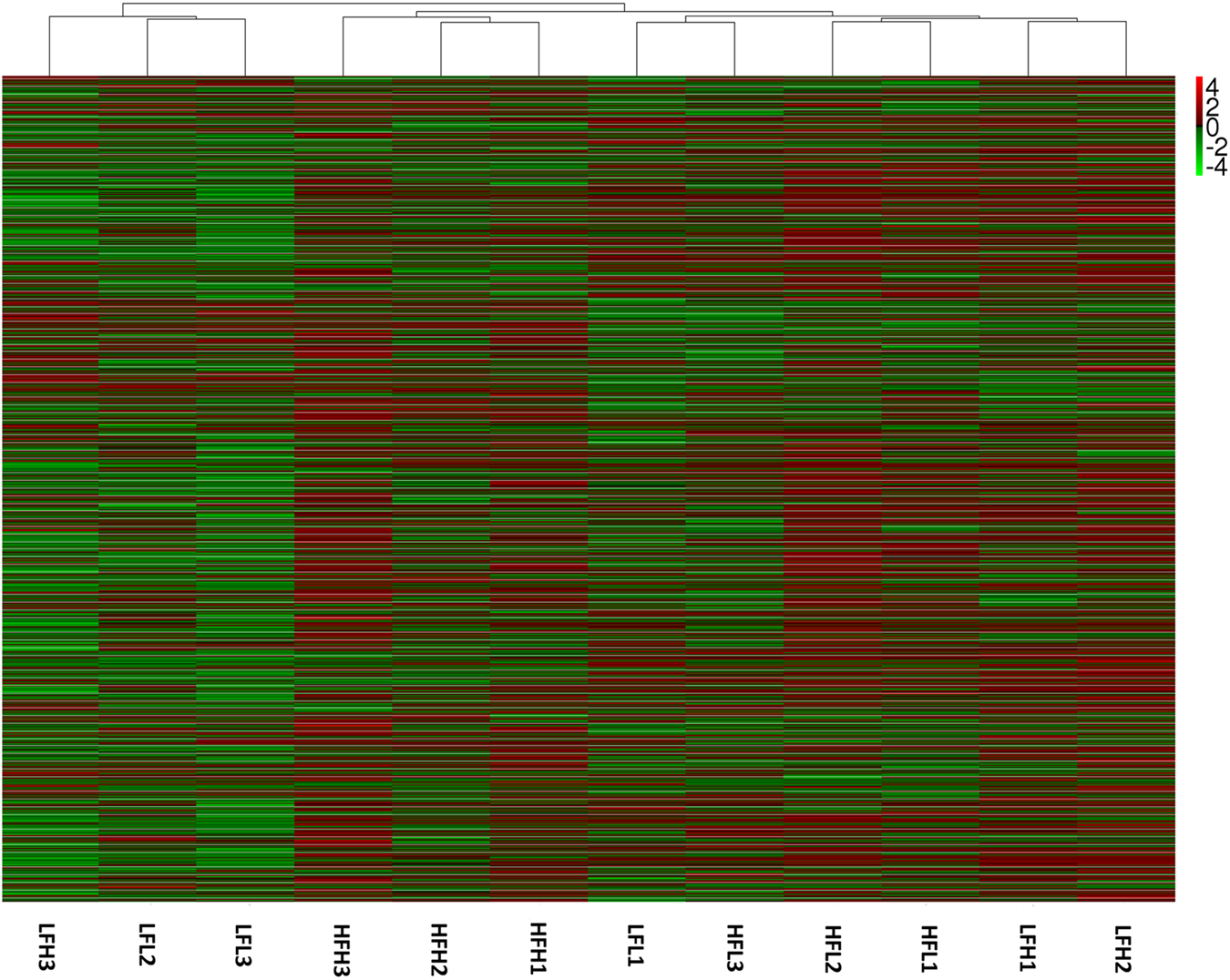
Hierarchical clustering of the 12 individuals on basis of the full gene expression profile. The red color represents the up expression, and the green color represents the down expression. The color from red to green represents the log10(RPKM+1) from large to small.

The expression levels of unigenes in each individual were estimated using RPKM (reads per kilobase of exon model per million mapped reads). A hierarchical clustering of the 12 individuals on basis of the full gene expression profile is shown in Fig. 2. Intriguingly, individuals within HF were not completely distinguishable from those within LF. There were also clear differences in gene expression pattern between the efficient and inefficient individuals within each family. As shown by the clustering patterns, 12 individuals were divided into two major groups, one including LFL2, LFL3 and LFH3, and the other including the remaining ones. Within the latter, HFH1, HFH2 and HFH3 were first grouped together, and then clustered with another subgroup that consisted of individuals from the two families. In this subgroup, a branch comprising LFH1 and LFH2, and another branch comprising HFL1 and HFL2 were clustered, and then they converged with a branch including HFL3 and LFL1. Apparently, the clustering patterns of HFL3 and LFL1 were inconsistent with their RFI distribution and family backgrounds, implying their abnormal expression patterns. As a result, HFL3 and LFL1 were excluded from the subsequent analysis. According to the RFI distribution and family backgrounds, the other 10 individuals were divided into a high-efficiency group (HG: HFH1, HFH2 and HFH3), a low-efficiency group (LG: LFL2, LFL3 and LFH3) and a medium control group (MG: LFH1, LFH2, HFL1 and HFL2). Of note is that there were no significant differences in ADG between three groups (*P* > 0.05).

### Identification of differentially expressed genes

To identify the genes associated with RFI, differentially expressed unigenes (DEGs) were determined by comparing their expression levels in each of HG and LG with those in MG. If a gene has a higher mRNA level in HG than in MG or in MG than in LG, it is considered as up-regulated, and conversely, it is down-regulated. Only those genes with both a false discovery rate (FDR) adjusted *P* value < 0.05 (*q* value < 0.05) and an absolute value of log2FoldChange ≥1 were considered to be significantly differentially expressed. As shown in Supplementary Table S2, 56 genes were differentially expressed in HG as compared with MG (*q* value < 0.05), among which 27 genes were down-regulated and 29 genes were up-regulated. All but one of the 56 DEGs between HG and MG were regulated more than five folds. Relatively, a large number of genes (348 genes) were differentially expressed between LG and MG (*q* value < 0.05), with 61 genes being down-regulated and 287 genes being up-regulated (Supplementary Table S3). Similarly, these genes were almost regulated five folds or more. There were 383 nonredundant genes between the two sets of DEGs, which were considered to be associated with RFI. These genes are mostly involved in cell proliferation, growth and signaling, glucose homeostasis, energy and nutrients metabolism.

Differential expression analysis between HG and LG was also performed to evaluate the influence of family genetic backgrounds on identification of DEGs. As shown in Supplementary Table S4, notably, a greater number of genes (946 genes) exhibited significantly differential expression between HG and LG (*q* value < 0.05), among which 617 genes had a higher expression in HG than in LG (up-regulated) and 329 genes were less expressed in HG than in LG (down-regulated). Interestingly, only 196 of these 946 genes matched to the putative RFI-associated genes.

### Functional enrichment analysis of differentially expressed genes

**Figure 3:**
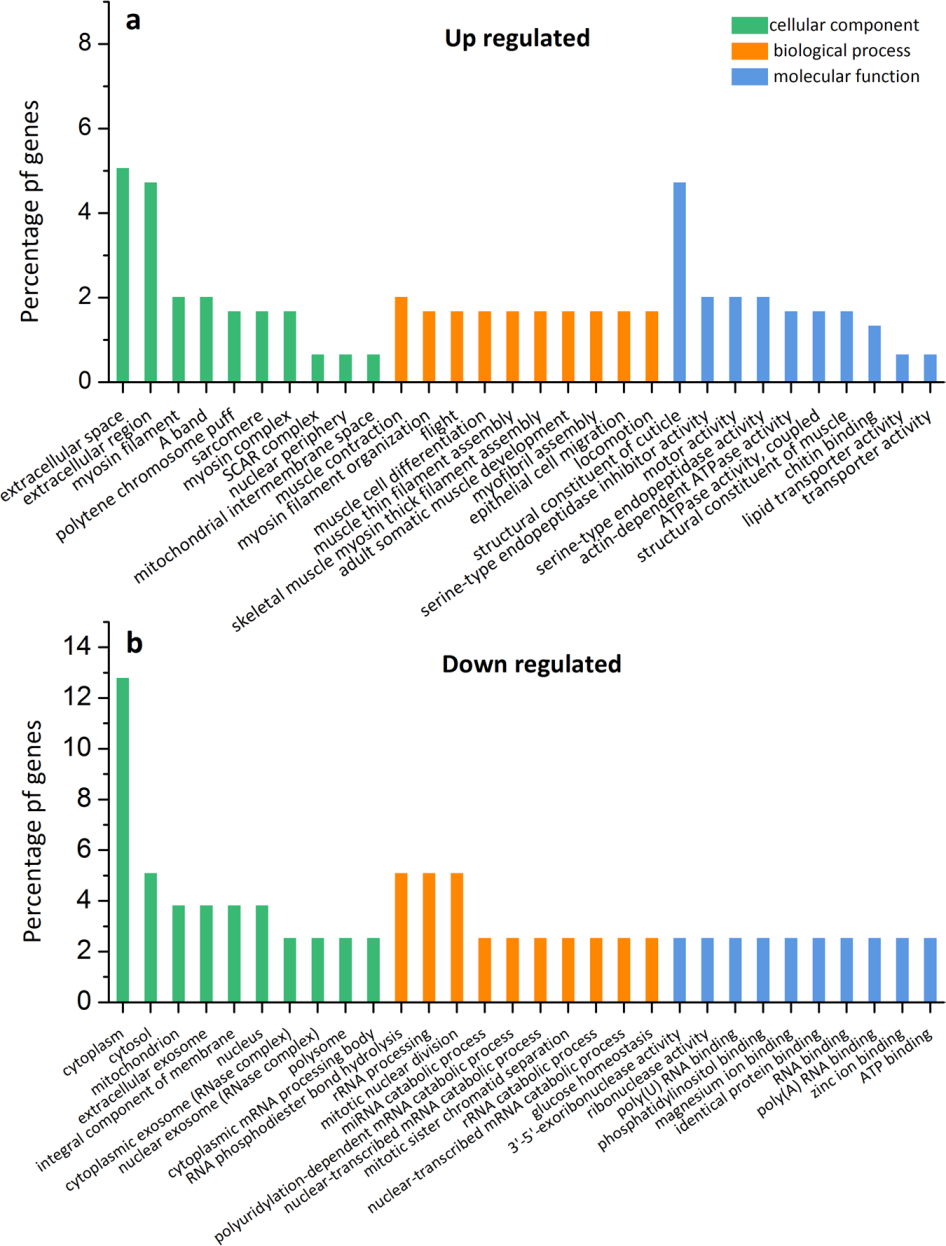
The top 10 significantly enriched GO terms in each of biological process, cellular component and molecular function for (**a**) the up-regulated differentially expressed genes and (**b**) the down-regulated differentially expressed genes, respectively. The x-axis represents GO terms belonging to three categories, and the y-axis represents gene percentages of each term.

To find out the function of DEGs and to analyze the potential biological pathways related to RFI, GO enrichment and KEGG pathway enrichment were performed for the 383 DEGs. In general, GO enrichment displayed 422 significantly enriched GO terms (*q* value < 0.05), containing 279 terms for biological progress, 48 terms for cellular component, and 95 terms for molecular function (see Supplementary Table S5). In addition, GO enrichment of the up-regulated and down-regulated DEGs are shown in Fig. 3a and b, respectively, which include the top 10 significantly enriched GO terms in each of “biological process”, “cellular component” and “molecular function” (*q* value < 0.05). Large differences in GO terms of each major category were seen between the up-regulated and down-regulated DEGs. Take “biological process” for example, the most enriched terms for the up-regulated DEGs were involved in “muscle function” and “development”, while those for the down-regulated DEGs were related to “nuclear division” and “RNA metabolic process”.

As shown in Table 2, there were 13 significantly enriched KEGG pathways (*q* value < 0.05). A large proportion of these pathways were involved in metabolism of nutrients, such as carbohydrate, cofactors and vitamin, and amino acid, among which amino sugar and nucleotide sugar metabolism (ko: 00520) and folate biosynthesis (ko: 00790) were the two most representative pathways. Several intuitively important signaling pathways were also found, such as PI3K-Akt signaling pathway (ko: 04151), AMPK signaling pathway (ko: 04152) and mTOR signaling pathway (ko: 04150). Additionally, there were some important pathways including ubiquitin mediated proteolysis (ko: 04120), cell cycle (ko: 04111), pentose phosphate pathway (ko: 00030) and glycolysis/gluconeogenesis (ko: 00010).

**Table 2:** The detailed information of 13 significant KEGG pathways enriched of the differentially expressed genes.

ID	Term	Number of genes	*q*-value	Gene ID
ko00790	Folate biosynthesis	4	8.11E-06	CL3954Contig2; CL7637Contig1; CL3673Contig1; CL17773Contig1
ko00520	Amino sugar and nucleotide sugar metabolism	5	2.95E-04	comp33199_c0_seq1_4; CL7654Contig1; CL3572Contig2; CL10922Contig1; CL140Contig9
ko00030	Pentose phosphate pathway	2	6.44E-03	CL25562Contig1; CL3572Contig2
ko00350	Tyrosine metabolism	1	2.50E-02	CL5946Contig1
ko00760	Nicotinate and nicotinamide metabolism	1	2.69E-02	CL51437Contig1
ko04111	Cell cycle - yeast	2	2.75E-02	CL698Contig2; comp23842_c0_seq2_2
ko00010	Glycolysis/Gluconeogenesis	2	2.85E-02	CL3572Contig2; CL10189Contig1
ko04151	PI3K-Akt signaling pathway	4	3.12E-02	CL17710Contig1; comp27190_c0_seq1_1; CL82Contig1; CL4000Contig1
ko00603	Glycosphingolipid biosynthesis - globo series	1	3.90E-02	CL7654Contig1
ko04120	Ubiquitin mediated proteolysis	2	3.92E-02	CL9297Contig2; comp31746_c1_seq2_1
ko04150	mTOR signaling pathway	1	4.05E-02	comp27190_c0_seq1_1
ko04152	AMPK signaling pathway	2	4.83E-02	CL13146Contig2; CL2118Contig3
ko00630	Glyoxylate and dicarboxylate metabolism	1	4.84E-02	CL9053Contig1

### Validation of expression levels using real time quantitative PCR

**Figure 4:**
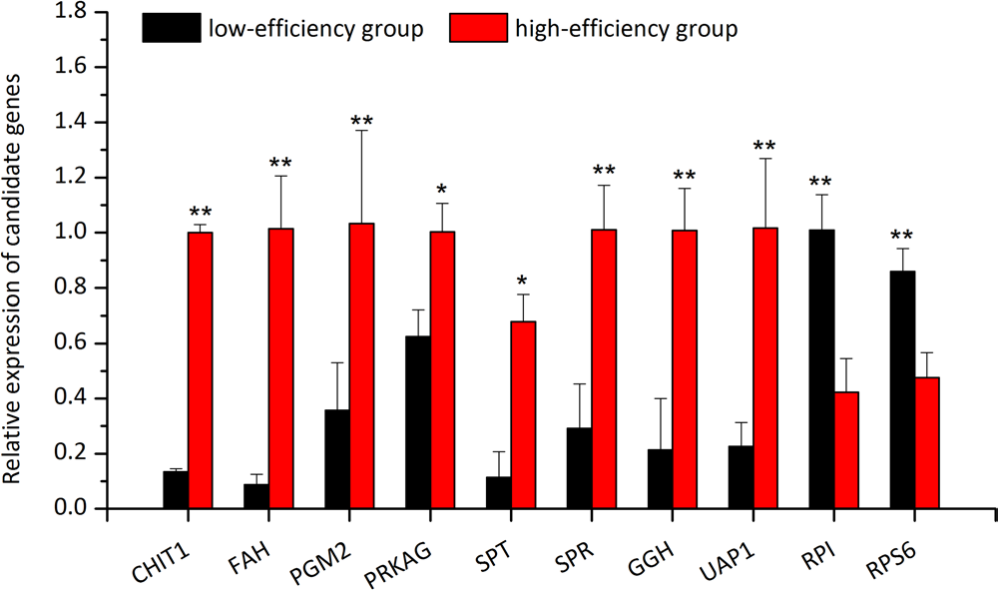
Expression pattern validation of 10 differentially expressed genes in a high-efficiency group and a low-efficiency group by real time quantitative PCR. *CHIT1*, *chitotriosidase-1* (comp33199_c0_seq1_4); *RPI*, *ribose-5-phosphate isomerase-like* (CL25562Contig1); *FAH*, *fumarylacetoacetase-like* (CL5946Contig1); *PGM2*, *phosphoglucomutase-2* (CL3572Contig2); *PRKAG*, *AMP-activated protein kinase subunit gamma* (CL13146Contig2); *RPS6*, *ribosomal protein S6* (comp27190_c0_seq1_1); *SPT*, *serine-pyruvate aminotransferase* (CL9053Contig1); *SPR*, *sepiapterin reductase* (CL3673Contig1); *GGH*, *gamma-glutamyl hydrolase-like isoform* × *1* (CL17773Contig1); *UAP1*, *UDP-N-acetylhexosamine pyrophosphorylase-like isoform* × *3* (CL10922Contig1).

To validate the expression patterns of the DEGs revealed by RNA-seq data, the relative expression levels of 10 important candidate genes in two validation groups were determined by real time quantitative PCR (RT-qPCR). As shown in Fig. 4, eight genes showed significantly higher expression levels in the high-efficiency validation group than in the low-efficiency validation group (*P* < 0.05), and two genes were significantly less expressed in the high-efficiency validation group than in the low-efficiency validation group (*P* < 0.05). Their quantitative results were in very good agreement with those revealed by RNA-seq, which confirms the reliability of RNA-seq and accuracy of DEG filtering process.

## Discussion

This study is the first attempt to identify RFI-associated genes in shrimp based on the global gene expression profiling, which is crucial to understand the genetic basis of RFI at the molecular level. We acquired more than 50 million reads from each sample sequenced, and performed a pooled transcriptome assembly from about 680 million reads of multiple samples and tissues. More than 70,000 unigenes were obtained with an average length of 1484 bp and N50 of 2481 bp, which appears robust when compared with some published data (Lu *et al*., 2016; Ghaffari *et al*., 2014; Guo *et al*., 2013)^21, 25, 26^. The large dataset allowed us to get an accurate reflection of transcript abundance for the differential expression analysis. However, only about a third of the unigenes have protein annotations, and much less genes are annotated in GO and KEGG database partly because of lacking the whole genome information of this species.

Although comparison of expression patterns between phenotypic extremes allows to identify genes associated with feed efficiency, a proper experiment design is crucial to this study considering the likely false positives caused by the differences in family genetic backgrounds. To solve this, we set up two groups with extreme phenotypes (HG and LG) and a control group based on both the RFI distribution and family backgrounds of the individuals used. Since the control group consisted of individuals from both HF and LF, we can consider that the genes differentially expressed between the extreme groups and the control group were not affected by family genetic backgrounds. Furthermore, the clustering patterns of individuals on basis of the full gene expression profile were checked to ensure the reliability of grouping. Two individuals (HFL3 and LFL1) whose clustering patterns were not in accordance with their RFI distribution and family backgrounds have not been considered in grouping. For differential expression analysis, using multiple individuals within a group as biological replicates could distinguish the genetic differences between groups from individual variation. In this study, 383 DEGs were identified to be associated with RFI, some of which has also been validated by the RT-qPCR experiment. Interestingly, a severe influence of family genetic backgrounds on expression profiles has been demonstrated, and only about 20% of the genes differentially expressed between HG and LG were associated with RFI. Therefore, direct comparison between families has proven to be not a good choice for the differential expression analysis.

Among the 383 putative RFI-associated genes, only 220 were annotated in the Nr database. Most of the genes with known function are involved in biological pathways related to cell proliferation, growth and signaling, glucose homeostasis, protein degradation, energy metabolism, carbohydrate metabolism, cofactors and vitamin metabolism, and amino acid metabolism. A previous study on cattle has reported 161 DEGs between the animals with high RFI and low RFI and seven gene networks involved in cellular growth and proliferation, cellular assembly and organization, cell signalling, protein synthesis, lipid metabolism, carbohydrate metabolism, and drug metabolism^13^. Another study that analyzed biological networks and pathways associated with RFI in beef cattle has also revealed some significant biological processes including lipid and steroid biosynthesis, protein and carbohydrate metabolism and regulation of gene expression through DNA transcription, protein stability and degradation^27^. Apparently, although species differences indeed exist in the determinants of RFI variation, some important biological processes appear to be consistent across species.

Based on KEGG enrichment analysis, a small number of the RFI-associated genes have further revealed some important biological pathways and mechanisms potentially responsible for RFI variation. During the grow-out period of shrimp, most of assimilated energy is channeled into metabolism maintenance and growth, and variation from them could possibly affect RFI phenotype. Growth of shrimp that concentrates in muscle tissue is largely a function of changes in cell number, size and metabolic activity. Consequently, the significant cell cycle pathway enriched of two DEGs in this study may have a relationship with the variation in growth components. With regard to muscle tissue that is the main place for protein storage as well as the main pool of amino acids in crustaceans^28, 29^, growth means protein synthesis in excess of catabolism, while metabolism means a balanced rate of catabolism and re-synthesis of protein. An earlier report has noted that protein turnover is responsible for a large fraction of the energy budget^30^. Greater levels of protein catabolism have been reported in cattle with high RFI than in cattle with low RFI^31^, which provides possible evidence for greater protein turnover in inefficient animals. As an important part of protein turnover, the pathway of ubiquitin mediated proteolysis involving two DEGs was significantly enriched in this study (Table 2), which may contribute to the variation in RFI.

Additionally, glucose metabolism in muscle has been reported to allocate energy for osmoregulation and physical activities in shrimp^32^. In this process, *L. vannamei* may first use glycogen as energy in muscle, and then use amino acids in muscle through gluconeogenesis^32^. In our results, the pathway of glycolysis/gluconeogenesis was enriched. It is inferred that some change of gene expression in this pathway may affect the utilization efficiency of energy in muscle. A gene encoding phosphoglucomutase-2 (CL3572Contig2) was found to be significantly up-regulated in this pathway (*q* value < 0.05). The product of this enzyme can enter glycolytic pathway for generating energy or enter the pentose phosphate pathway for generating biosynthetic intermediates. This gene also works in pentose phosphate pathway that has been reported to be involved in carbohydrate metabolism in decapods during ecdysis^33^.

The AMP-activated protein kinase (AMPK) has an important role in the regulation of cellular energy homeostasis. AMPK on the one hand inhibits synthesis of fatty acids, cholesterol, and triglycerides, and activates fatty acid uptake and β-oxidation by phosphorylating acetyl-CoA carboxylase 1 or sterol regulatory element-binding protein 1c^34, 35^. It on the other hand stimulates glucose uptake in skeletal muscle and stimulates glycolysis, and inhibits glycogen synthesis^36–38^. AMPK is regulated allosterically mostly by competitive binding on its gamma subunit between ATP, AMP or ADP^39^. Low energy states, represented by a high AMP/ATP ratio, results in increased AMPK activity and suppression of mTOR-mediated growth pathways^40^. In mammals, the effect of AMPK on energy balance could extend to whole-body energy homoeostasis by integrating nutritional and hormonal signals that control food intake and body weight in the hypothalamus^41^. It has been reported that AMPK signaling pathway is involved in the variation of RFI in cattle^27^. As shown in our results, a gene (CL13146Contig2) annotated as AMPK subunit gamma was significantly up-regulated (*q* value < 0.05), which is likely to have an influence on the variation in RFI.

In mammals, mTOR signaling seems to have a prominent role in the molecular control of feeding behavior, and some studies showed that overactivation of mTOR in catabolic pro-opiomelanocortin neurons reduced their activity and resulted in disinhibition of feeding and obesity^42, 43^. However, it is not clear whether mTOR signaling has the similar effect in shrimp. mTOR signaling can also be regulated by AMPK via multiple cellular mechanisms. In this study, expression of ribosomal protein S6 (comp27190_c0_seq1_1) in the downstream processes of mTOR signaling was significantly down-regulated (*q* value < 0.05). This gene is thought to be an effector in the regulation of cell size, cell proliferation, and glucose homeostasis^44^. As another important pathway, PI3K-Akt signaling pathway activated by various cellular stimuli or toxic insults can regulate fundamental cellular functions such as transcription, translation, proliferation, growth, and survival. There were four genes enriched in this pathway, among which three up-regulated genes (CL17710Contig1, CL82Contig1 and CL4000Contig1) matched to the extracellular matrix (ECM). It seems plausible to suggest that these up-regulated genes are likely to enhance the PI3K/AKT signaling pathway by stimulating PI3K. Then PI3K can regulate a cascade of changes involved in apoptosis, protein synthesis, metabolism, and cell cycle^45, 46^.

In summary, some genes and related biological pathways were identified to be associated with RFI in this study, which provides initial insight into the molecular mechanisms driving the feed efficiency in *L. vannamei*, and ﻿provides ﻿potential targets for molecular breeding efforts to develop high-efficiency variety of *L. vannamei*. As expected, no single mechanism can be primarily responsible for RFI variation, and there appears close ties among some biological pathways. Further research on the relationships among the RFI-associated genes and pathways is necessary to elucidate the detailed molecular mechanisms related to the RFI variation. Besides, more well-characterized populations of animals that have been reliably phenotyped for RFI are desired instead of families for more advanced molecular and genetic studies.

## Methods

### RFI data

This research was approved by the Animal Care and Use committee in the Yellow Sea Fisheries Research Institute, Chinese Academy of Fishery Sciences. All experiments complied with the Law of the People’s Republic of China on the Protection of Wildlife (http://www.china.org.cn/english/environment/34349.htm). The breeding population of *L. vannamei* was established in 2011, and since then the closed generation has been produced yearly^47^. The breeding work was finished at the Mariculture Research Station of Yellow Sea Fisheries Research Institute, Chinese Academy of Fishery Sciences (Qingdao, China).

In 2015, 33 families with high survival were used for a feeding test. Thereinto, 18 individuals were randomly sampled from each family when the smallest shrimp reached 4 cm. The feeding test was conducted in the aquatic housing systems (Haisheng Biotech, Shanghai, China) that were constituted of many independent culture tanks. Each individual was reared in one tank and fed with formulated pellet diets (Haid Dachuan #2, Guangdong Haid, China) three times every day, at 9:00, 16:00 and 23:00, respectively. An appropriate feed dose per meal was ensured for their apparent satiation. For each shrimp, unconsumed feed was collected into an independent container every day and then dried until weight was stable. The test continued for three weeks. Body weight at the start (BW1) and end (BW2) of the experiment, body weight gain (WG) and feed intake (FI) were recorded for each shrimp, and dead animals and those with obviously unreasonable data were removed from subsequent analysis. ADG and DFI were obtained as BW and FI divided by 21 days, respectively. To determine RFI, expected feed intake was calculated as a multiple regression with observed feed intake as the dependent variable^48^:

1 $${\rm{DFI}}={\rm{b}}1\times {{\rm{MW}}}^{{\rm{b}}2}+{\rm{b}}3\times {\rm{ADG}}+{\rm{e}}$$

where MW is the mid-weight (MW = 1/2(BW1 + BW2)), MW^b2^ is the metabolic mid-weight, DFI and ADG are described as above, e is the error, and b1, b2, b3 are partial regression coefficients. The error term is considered as RFI. The calculation was finished using the nonlinear regression procedure of nls in R 3.3.1 software^49^.

### Sampling, RNA isolation and Illumina sequencing

The final dataset included 506 animals, with 11–18 individuals per family. Based on the average values of RFI, we chose the family with the highest efficiency and that with the lowest efficiency. From each family, we collected the three most efficient individuals (HFH1, HFH2 and HFH3 for HF, and LFH1, LFH2 and LFH3 for LF) and the three least efficient individuals (HFL1, HFL2 and HFL3 for HF, and LFL1, LFL2 and LFL3 for LF).

Muscle tissue of the third abdominal segment was sampled from each of the 12 individuals at half an hour after a feed. In order to obtain a better *de novo* assembly, the whole hepatopancreas tissue of an individual (HFH1) was also sampled for a pooled transcriptome assembly together with the muscle samples. All tissue samples were ground separately to a fine powder in the presence of liquid nitrogen. Total RNA was extracted using the TRIzol Reagent (Invitrogen, USA) and treated with DNase I. Quality and abundance of RNA were verified using a Bioanalyzer 2100 (Agilent Technologies, USA).

Sequencing libraries were constructed using Illumina TruSeq RNA Sample Peparation Kit (Illumina, USA), as dictated by the TruSeq protocol. The libraries were amplified with 15 cycles of PCR and then sequenced on the Illumina HiSeq^TM^ 2500 platform (Illumina, USA) with 125 bp paired-end reads. The image analysis, base calling and quality score calibration were processed using Illumina Pipeline Software v1.5, and FASTQ reads files containing the sequencing read, quality scores and paired reads information were exported for the following trimming and assembly process. The adapter sequences, low quality reads (quality scores <30 or read length <30 bp), and reads with poly-N were removed from the raw reads, and the high quality reads were used for the downstream analysis.

### *De novo* assembly and annotation

The trimmed and quality-filtered sequences from 12 muscle samples and one hepatopancreas sample were used for assembly in Trinity software^50^. Unigenes were identified as the longest transcripts for each gene to avoid redundant transcripts. All assembled unigenes were used as queries against NCBI Nr database (http://www.ncbi.nlm.nih.gov/) and Swiss-Prot database (http://www.ebi.ac.uk/uniprot/) using BlastX with E-values ≤ 1E-5. In addition, to have a more comprehensive understanding of unigenes expressed in the target tissue, GO (http://www.geneontology.org/) and KEGG classification (http://www.genome.jp/kegg/) were performed with Blast2GO^51^ and KOBAS 2.0 software^52^, respectively.

### Cluster analysis, grouping, and differential expression analysis

Read count values of unigenes in each individual were obtained by mapping clean data back onto the transcripts using HTSeq v0.6.0. RPKM was calculated to estimate the expression levels of unigenes for each individual^53^. Hierarchical clustering of the 12 individuals was performed using the full gene expression profile and clusters were extracted using R scripts.

Since DEGs identified through comparison between HF and LF may include false positives caused by different family genetic backgrounds, we used another strategy for differential expression analysis. To be specific, we set up two groups with extreme RFI phenotypes (HG and LG) and a medium control group (MG) based on the RFI distribution and family backgrounds of the 12 individuals. HG comprised the most efficient individuals in HF, LG comprised the least efficient individuals in LF, and MG was consisted of the remaining individuals in both HF and LF. Importantly, optimal grouping was determined according to the clustering patterns of individuals, and individuals with obvious abnormal expression patterns were excluded.

Differential expression analysis was performed between HG and MG, between MG and LG, and between HG and LG, respectively. Individuals from each group can be regarded as biological replicates and DEGseq R package was used to determine DEGs between groups. To improve the credibility of DEGs, the initial *P* value were adjusted by the FDR method^54^. The expression fold change was calculated between groups. DEGs with a FDR adjusted *P* value < 0.05 and an absolute value of log2FoldChange ≥1 were considered as significant.

### GO and KEGG enrichment analysis

GO enrichment analysis was implemented for all DEGs, up-regulated DEGs and down-regulated DEGs, respectively, by Blast2GO^49^, with gene length bias being corrected. GO terms with *q* value < 0.05 were considered to be significantly enriched. To explore the biological pathways and molecular mechanisms related to RFI, the enrichment of all these DEGs in KEGG pathways was statistically tested with KOBAS software^50^, at a significant level of *q* value < 0.05.

### Validation of expression levels using RT-qPCR

To validate the results of differential expression analysis from RNA-seq data, the expression patterns of 10 important DEGs were detected by RT-qPCR technology in other animals. The animals were collected from another independent family according to the RFI values, constituting a high-efficiency validation group and a low-efficiency validation group, respectively, each containing three individuals. RNA samples and cDNA were prepared using the same method mentioned above. The specific primers were designed using the Primer Premier 5 software (Premier Biosoft International, USA) according to Illumina sequencing data. The 18S gene of *L. vannamei* was used as an internal control to normalize the expression level. The primer sequences are listed in Supplementary Table S6. Samples were run in technical triplicate on an ABI 7500 Real-time PCR System (Applied Biosystems, USA) following the manufacturer’s instructions, and the detailed description of RT-qPCR was previously reported^21^. The relative expression levels were calculated by the comparative 2−ΔΔCt method^55^.

## Electronic supplementary material

Dataset 1

Dataset 2

Dataset 3

Dataset 4

Dataset 5

Dataset 6
